# In vitro fertilization results of GNRH antagonists and medroxyprogesterone acetate used to prevent premature LH surge during ovarian hyperstimulation

**DOI:** 10.1038/s41598-024-67280-7

**Published:** 2024-07-12

**Authors:** Can Dinç, Saltuk Buğra Arıkan, Mustafa Özer, Şafak Olgan

**Affiliations:** Department of Gynecology and Obstetrics, Akdeniz University, 07010 Antalya, Turkey

**Keywords:** Ovarian stimulation, Premature LH surge, Medroxyprogesterone acetate, Clinical pregnancy rates, Infertility, Endocrine reproductive disorders

## Abstract

The aim of this study was to evaluate the effects of medroxyprogesterone acetate (MPA) treatment in comparison to those of gonadotropin releasing hormone (GnRH) antagonists for the prevention of premature luteinizing hormone surges during controlled ovarian hyperstimulation (OS) and the impact of these effects on developing embryos and pregnancy outcomes. Data from 757 cycles of GnRH antagonist treatment and 756 cycles of MPA treatment were evaluated at the Akdeniz University Faculty of Medicine Assisted Reproductive Treatment Center between October 2018 and April 2022. Patient records were obtained from the electronic database of the centre and analysed. In our centre, GnRH antagonist protocols were used between 2018 and 2020, and MPA protocols were used between 2020 and 2022. We chose our study population by year. Our study is a comparative retrospective study. All methods in this study were performed in accordance with the relevant guidelines and regulations. Patients using MPA were significantly older (33.9 ± 5.6 vs. 32.6 ± 5.6, *p* < 0.001) and had a lower number of antral follicles (AFC) (10.7 ± 8.6 vs. 11.9 ± 10.8, *p* = 0.007) than those using GnRH antagonists. Both MPA (2.9%) and GnRH antagonists (2.2%) had similar effectiveness in preventing premature ovulation (*p* = 0.415). There was no significant difference between the two groups in terms of the number of total developed embryos (1.3 ± 1.3 vs. 1.2 ± 1.2, *p* = 0.765). There was no significant difference in the clinical pregnancy rates with the first ET (%35.4 vs. %30.1, *p* = 0.074), per total number of transfers (35.3% vs. 30.1%, *p* = 0.077). MPA was found to be effective at preventing premature ovulation during OS treatment, and the incidence of developing embryo and pregnancy outcomes in patients using MPA were similar to those in patients using GnRH antagonists. Therefore, the use of MPA instead of GnRH antagonists during OS may be a viable alternative for patients not scheduled for fresh ET.

## Introduction

The occurrence of a premature LH surge of up to 20% in OS cycles negatively affects pregnancy outcomes. With the introduction of gonadotropin-releasing hormone analogues (GnRHa), a premature LH surge has been prevented, and pregnancy rates have increased^1^.

The main methods used to prevent a premature LH surge are the use of GnRH agonists or GnRH antagonists during ovarian stimulation^2^. Both of these GnRH analogues have disadvantages such as increased ovarian hyperstimulation syndrome (OHSS), high cost, and procedure complexity^3^.

Therefore, the search for new methods to inhibit the premature LH surge is still ongoing. There is already strong evidence that progestin formulations in oral contraceptive (OC) therapies effectively prevent LH surges and ovulation. Therefore, progestins are considered by reproductive endocrinologists to be a suitable alternative to conventional GnRH-analogue therapy for ovarian stimulation for IVF^4^.

In the past, when frozen embryo transfer (ET) was not widely used, progestins were not considered for use during ovarian stimulation (OS) because they are known to have a negative effect on endometrial receptivity. However, with advances in embryo vitrification technologies, fresh ET is no longer a necessary step. There is also increasing evidence that freeze–thaw ET may result in similar pregnancy and live birth outcomes^5,6^.

Synthetic progestins can be used as alternatives to GnRH analogues to prevent premature LH surges during OS in in-vitro fertilization (IVF) cycles due to their LH suppression activity and freeze-all approach^7^.

The aim of this study was to compare the frequency of premature ovulation, the number of oocytes obtained, the number of high-quality embryos developed, and the cumulative pregnancy outcomes after fresh or frozen ETs from these embryos in patients treated with MPA and GnRH antagonists to prevent a premature LH surge.

## Materials and methods

Patients who underwent OS between October 2018 and April 2022 at the Akdeniz University Faculty of Medicine Assisted Reproductive Treatment Center and who received GnRH antagonists or MPA to prevent premature LH surges were included in the study. Approval for the study was obtained from the Akdeniz University Faculty of Medicine Ethics Committee on 18.08.2021 (KAEK-588).

### Allocation and sample size estimation

Overall, 1513 OS cycles were performed during the study period. GnRH antagonists were used in 757 cycles, and MPA was used in 756 cycles to prevent a premature LH surge. In our centre, GnRH antagonists were used between 2018 and 2020, and MPA was used between 2020 and 2021. We chose our patients by year. Our study is a comparative retrospective study.

### Procedures

Basal FSH, LH, estradiol (E2), thyroid stimulating hormone (TSH) and prolactin measurements were performed on the 2nd-3rd day of menstruation in patients to be started on OS, and basal endometrial thickness and total antral follicle count (AFC) in bilateral ovaries were evaluated by transvaginal ultrasound (TVUSG). If E2 was < 60 pg/ml and no dominant follicle was found on TVUSG, gonadotropin stimulation was started. The starting dose of Gn was between 150 and 450 IU according to the patient's age, ovarian reserve, body mass index (BMI) and follicle development during the previous OS. The gonadotropins used were human menopausal gonadotropin (hMG) (Merional, 150 IU im/sc, IBSA Institut Biochimique, Switzerland or Menopur 150 IU im/sc, Ferring GmbH, Germany), recombinant FSH (rFSH) (Gonal-f 450 IU/0.75 ml Merck Serono, Italy) or both hMG and rFSH. GnRH antagonists (Cetrotide 250 mcg sc, Pierre Fabre Medicament Production, France) were added to the fixed regimen on Day 5 of stimulation. In the MPA cycles, MPA (Tarlusal 5 mg, 2 × 1 oral, Deva Holding, Turkey) was added to the treatment beginning on the first day of gonadotropin stimulation. Patients who received both the GnRH antagonist and MPA were called for USG control on the 5th day of stimulation, and the number and size of developing follicles were recorded. Serum FSH, LH, E2, and progesterone measurements were repeated for all controls. The number and size of follicles that developed during the follow-ups were evaluated, the dose of gonadotropin used was adjusted, and the frequency of the next follow-up was determined. After the follicles reached 17–19 mm in size (≥ 2 follicles), ovulation was induced with human chorionic gonadotropin (hCG) (Ovitrelle, 250 µg, Merck Serono, Italy) and/or triptorelin acetate (Gonapeptyl, 0.2 mg Ferring GmbH, Germany).

Oocyte pick-up (OPU) was performed 34–36 h after ovulation was triggered.

Embryos that reached the blastocyst stage were analysed using the ALPHA Istanbul consensus evaluation system^8^.

In cycles in which GnRH antagonists were administered, ET was performed on Days 4–6 after OPU if there was no obstacle to fresh ET. However, for patients with OHSS risk or elevated progesterone levels on the day of hCG, all embryos were frozen, and fresh ET was not performed. In the MPA cycles, all developing embryos were frozen, and frozen ET was performed 1 month later by artificially preparing the endometrium with hormone replacement therapy (HRT). If the beta hCG result was > 20 mIU/mL and an increase was observed in the control two days later, it was considered a positive hCG. The patients in which pregnancy was detected were called for TVUSG control after 2 weeks. A decrease in the beta hCG value without any intrauterine gestational sac was considered a biochemical pregnancy. A fetal heartbeat on USG 6 weeks after the transfer was considered a clinical pregnancy. We do not have the data on baby delivery rates.

### Statistical analysis

The data collected in this study were analysed using the Statistical Package for the Social Sciences 25.0 (SPSS 25.0). The mean ± standard deviation was calculated for descriptive statistics of the data. In cases where the univariate normal distribution assumption was met, the unpaired samples t test was used to evaluate the significance of the difference between two unrelated sample means. In cases where this assumption was not met, the Mann–Whitney U test, the nonparametric equivalent of the unpaired samples t test, was used. The chi-square test was used to determine whether the relationship between two classified variables was significant. Fisher’s exact test was used if the chi-square test was 2 × 2 and the expected value of one of the pores was less than 5; in other cases, the p value for Pearson chi-square was taken into consideration. The significance level was accepted as *p* < 0.05 in the analyses.

### Ethics approval

Approval for the study was obtained from the Akdeniz University Faculty of Medicine Ethics Committee on 18.08.2021 (KAEK-588).

### Consent to participate

Written informed consent was obtained from all patients.

## Results

Overall, 1513 OS cycles were performed during the study period. GnRH antagonists were used in 757 cycles, and MPA was used in 756 cycles to prevent a premature LH surge.

The demographic characteristics of the patients who received GnRH antagonists and MPA are shown in Table 1.

**Table 1:** Demographic, clinical and laboratory data of patients of GnRH antagonist and MPA groups.

Variabilities		GnRH antagonist (n = 757)	MPA (n = 756)	*P* value
Age (mean ± SD)		32,6 ± 5,6	33,9 ± 5,6	$<0{,001}^{\text{a}}$
Body mass index (BMI) ($\text{kg}/{\text{m}}^{2}$)		25,3 ± 5.1	25,1 ± 4,8	$0{,863}^{\text{b}}$
Duration of infertility (year),		4,1 ± 3,5	3,4 ± 3,0	${\text{0,001}}^{\text{b}}$
Chronic comorbidities*		23,5 (178)	25,4 (192)	$0{,394}^{\text{c}}$
Total progressive motile sperm count (TPSC) (mean ± SD)		46,59 ± 60,7	25,11 ± 39,0	$<0{,001}^{\text{b}}$
Antral follicle count (AFC) (mean ± SD)		11,97 ± 10,8	10,72 ± 8,6	$0{,007}^{\text{b}}$
Basal estradiol (E2) (mean ± SD)		60,12 ± 51,0	68,88 ± 81,4	$0{,850}^{\text{b}}$
Basal luteinizing hormone (LH) (mean ± SD)		5,16 ± 3,8	5,84 ± 5,0	$0{,012}^{\text{b}}$
Basal FSH (mean ± SD)		7,97 ± 5,2	8,40 ± 5,9	$0{,605}^{\text{b}}$
Follicle-stimulating hormone (FSH) starting dose (day) (mean ± SD)		208,6 ± 61,0	276,5 ± 44,9	$<0{,001}^{\text{b}}$
Duration of stimulation (day)		9,5 ± 2,2	9,1 ± 2,0	$<0{,001}^{\text{b}}$
Total FSH dose (mean ± SD)		3009,6 ± 1356,6	2685,8 ± 852,7	$0{,001}^{\text{b}}$
Gonadotropin type	Human menopausal gonadotropin HMG	%28,8 (218)	%11,1 (84)	$<0{,001}^{\text{b}}$
	Recombinant (Rec.) FSH	%4,1 (31)	%77,6 (587)	
	Recombinant (Rec.) FSH + HMG	%67,1 (508)	%11,2 (85)	
Minimum LH (mean ± SD)		1,6 ± 1,6	4,3 ± 3,2	$<0{,001}^{\text{b}}$
Maximum LH (mean ± SD)		5,4 ± 3,9	8,1 ± 8,7	$<0{,001}^{\text{b}}$
hCG day E2 (mean ± SD)		2183,5 ± 5592,3	2516,1 ± 2069,2	$<0{,001}^{\text{b}}$
hCG day LH (mean ± SD)		3,1 ± 6,4	6,1 ± 10,1	$<0{,001}^{\text{b}}$
hCG day progesterone (mean ± SD)		0,8 ± 0,5	0,9 ± 1,4	$0{,397}^{\text{b}}$
Trigger type	hCG	%3,2 (24)	%1,6 (12)	$<0{,001}^{\text{b}}$
	GnRH agonistx2	%4,2 (32)	%12,2 (92)	
	hCG + GnRH agonist	%92,6 (701)	%86,2 (652)	
Total number of oocytes (mean ± SD)		10,2 ± 7,9	9,3 ± 8,1	$0{,004}^{\text{b}}$
Metaphase 2 (MII) oocyte count (mean ± SD)		7,1 ± 6,0	6,9 ± 6,2	$0{,218}^{\text{b}}$
2 pro-nuclei (2PN) count (mean ± SD)		3,5 ± 3,5	3,2 ± 3,2	$0{,289}^{\text{b}}$
Total number of embryos (mean ± SD)		1,2 ± 1,2	1,3 ± 1,3	$0{,765}^{\text{b}}$
High embryo quality		%32,4 (245)	%39,5 (298)	$<0{,001}^{\text{c}}$
Poor embryo quality		%39,9 (302)	%28,8 (218)	
MII oocytes/total oocytes (%) (mean ± SD)		0,6 ± 0,2	0,7 ± 0,2	${<\text{0,001}}^{b}$

*Chronic diseases: Asthma, Behçet’s disease, cardiac diseases, diabetes mellitus, endometriosis, familial Mediterranean fever, gasroenterological diseases, hypertension, liver diseases, malignancy, pituitary gland diseases, psychiatric diseases, renal diseases, rheumatoid arthritis, systemic lupus eriematosus, thalassemia, thyroid diseases, vaginismus.

^a^Unpaired samples t-test.

^b^Mann Whitney U-test.

^c^Pearson chi-square.

GnRH antagonist patients (32.6 ± 5.6) were significantly younger than those in the MPA group (33.9 ± 5.6) (*p* < 0.001). However, there was no significant difference between the mean BMI of the patients (*p* > 0,05). The mean duration of infertility was lower in the MPA group (3.4 ± 3.0) than in the GnRH antagonist group (4.1 ± 3.5) (*p* < 0.001). There was no significant difference in the frequency of chronic diseases between the MPA and GnRH antagonist groups (*p* = 0.394).

The mean total progressive motile sperm count (TPSC) in the GnRH antagonist group (46.5 ± 60.7 million) was significantly greater than that in the MPA group (25.1 ± 39.0 million) (*p* < 0.001). Similarly, the mean total antral follicle count (AFC) was greater in the GnRH antagonist group (11.9 ± 10.8) than in the MPA group (10.7 ± 8.6) (*p* = 0.007). The basal E2 (*p* = 0.850) and FSH (*p* = 0.605) levels were similar between the MPA and GnRH antagonist groups. However, the mean basal LH level in the MPA group (5.8 ± 5.0 IU/L) was greater than that in the GnRH antagonist group (5.1 ± 3.8 IU/L) (*p* = 0.012).

The mean daily FSH dose in the MPA group (276.5 ± 44.9 IU) was greater than that in the GnRH antagonist group (208.6 ± 61.0 IU) (*p* < 0.001). In contrast, the duration of ovarian stimulation was shorter in the MPA group (9.1 ± 2.0 days) than in the GnRH antagonist group (9.5 ± 2.2 days) (*p* < 0.001). Similarly, the mean total FSH dose in the MPA group (2685.8 ± 852.7 IU) was significantly lower than that in the GnRH antagonist group (3009.6 ± 1356.6 IU) (*p* < 0.001).

There was a significant difference between the types of gonadotropins used in the MPA and GnRH antagonist groups (*p* < 0.001). Most of the patients in the MPA group were treated with hMG alone (77.6%), whereas in the GnRH antagonist group (67.1%), hMG was used in combination with rFSH.

The mean minimum LH in the MPA group (4.3 ± 3.2 IU/L) was greater than that in the GnRH antagonist group (1.6 ± 1.6 IU/L) (*p* < 0.001). Similarly, a significant difference was observed between the mean maximum LH values measured during the cycle in the MPA and GnRH antagonist groups (*p* < 0.001).

There was a statistically significant difference in the type of triggering between the MPA and GnRH antagonist groups (*p* < 0.001). However, the majority of patients in both the MPA (86.2%) and GnRH antagonist (92.6%) groups were administered a GnRH agonist with hCG. We did not evaluate the number or quality of the embryos of patients where the final triggering was performed by GnRH agonist, hCG injections, and/or both.

The mean E2 concentration on the day of triggering in the MPA group (2516.1 ± 2069.2 pg/ml) was significantly greater than that in the GnRH antagonist group (2183.5 ± 5592.3 pg/ml) (*p* < 0.001). Similarly, the mean LH on the day of triggering in the MPA group (6.1 ± 10.1 IU/L) was significantly greater than that in the GnRH antagonist group (3.1 ± 6.4 IU/L) (*p* < 0.001). However, the progesterone levels measured on the day of triggering were not significantly different between the groups (*p* = 0.397).

The total number of oocytes obtained in the MPA group (9.3 ± 8.1) was lower than that in the GnRH antagonist group (10.2 ± 7.9) (*p* = 0.004). However, there was no statistically significant difference between the Metaphase 2 (MII) oocyte counts of the groups (*p* = 0.218). Similarly, no statistically significant difference was observed between the mean total 2 pro-nuclei (2PN) oocyte counts of the patients in the MPA and GnRH antagonist groups (*p* = 0.289).

The total number of developing embryos was similar between the groups (*p* = 0.765). However, the number of high-quality embryos that developed in the GnRH antagonist and the MPA groups was significantly different (*p* < 0.001), which was 39.5% and 32.4% for the MPA and the GnRH antagonist groups, respectively.

The ratio of MII to total oocytes in the MPA group (0.7 ± 0.2) was significantly greater than that in the GnRH antagonist group (0.6 ± 0.2).

In our study, an early LH surge was defined as a 1.8-fold increase in basal LH values during the OS cycle. There was no significant difference in the occurrence of a premature LH surge between the MPA and GnRH antagonist groups (*p* = 0.708). A premature LH surge was not observed in most of the patients in either the MPA or the GnRH antagonist groups. The rate of premature LH surge in the MPA group (19.3%) was lower than that in the GnRH antagonist group (20.1%).

When the maximum LH values of the patients were analysed, it was found that the frequency of LH values above 10 IU/L was significantly greater in the MPA group (19.8%) than in the GnRH antagonist group (9.0%) (*p* < 0.001). Similarly, when the maximum LH values of the patients were analysed, the frequency of LH values above 15 IU/L were significantly higher in the MPA group (6.1%) than in the GnRH antagonist group (3.6%) (*p* = 0.022). On the other hand, the frequency of premature ovulation was similar between the GnRH antagonist (2.2%) and MPA (2.9%) groups (*p* = 0.415).

The number of cycles with a 1.8-fold increase in the basal LH value or maximum LH values above 10 IU/L and 15 IU/L and the frequency of premature ovulation in the MPA and GnRH antagonist groups are shown in Table 2.

**Table 2:** Examination of cycles with 1.8-fold increase in basal LH value or maximum LH values above 10 IU/L and 15 IU/L measured in MPA and GnRH antagonist group.

	GnRH antagonist (n = 757)	MPA (n = 756)	*P* value
1.8-fold increase of LH during OS (%, n)	%20,1 (152)	%19,3 (146)	$0{,708}^{\text{a}}$
Maximum LH > 10 IU/L during OS (%, n)	%9,0 (68)	%19,8 (150)	${<\text{0,001}}^{\text{a}}$
Maximum LH > 15 IU/L during OS (%, n)	%3,6 (27)	%6,1 (46)	$0{,022}^{\text{a}}$
Premature ovulation (%, n)	%2,2 (17)	%2,9 (22)	${\text{0,415}}^{\text{a}}$

^a^Pearson chi-square.

Cycle cancellations were made for different reasons for 195 (25.6%) patients in the GnRH antagonist group and 239 (31.6%) patients in the MPA group. Cycle cancellation rates were similar between the GnRH antagonist and MPA groups (*p* = 0.244). Cycle cancellation due to embryo arrest (42.2%) was more common in the MPA group, while it was more common due to fertilization failure (35.8%) in the GnRH antagonist group. In our study, we did not evaluate OHSS rates.

The reasons for cycle cancellation after OS or OPU procedures are given in Table 3.

**Table 3:** Reasons for cycle cancellation after OS or OPU procedure.

Reasons for cycle cancellation	GnRH antagonist (n = 195)	MPA (n = 239)	*p* value
Failure to obtain oocytes in OPU (n, %)	23 (%11,8)	26 (%10,9)	$0{,244}^{\text{a}}$
Abnormal oocyte (n, %)	36 (%18,5)	45 (%18,8)	
Fertilization failure (n, %)	70 (%35,9)	67 (%28,0)	
Embryo arrest (n, %)	66 (%33,8)	101 (%42,3)	

There were no significant differences in the hCG positivity rate (*p* = 0.104), biochemical pregnancy rate (*p* = 0.713), or clinical pregnancy rate (*p* = 0.074) between the two groups after the first ET.

The pregnancy results of both groups in the first ET are given in Table 4.

**Table 4:** Pregnancy results of both groups in the first ET.

		GnRH antagonist (n = 535)	MPA (n = 466)	*p* değeri
Positive hCG (%, n)	None	%60,8 (325)	%55,8 (260)	$0{,104}^{\text{a}}$
	Yes	%39,2 (210)	%44,2 (206)	
Biochemical pregnancy (%, n)	None	%91,2 (488)	%91,2 (425)	$0{,713}^{\text{a}}$
	Yes	%8,8 (47)	%8,7 (41)	
Clinical pregnancy (%, n)	None	%69,9 (374)	%64,6 (301)	$0{,074}^{\text{a}}$
	Yes	%30,1 (161)	%35,4 (165)	

^a^Pearson Ki-Kare.

The cumulative pregnancy results per total number of transfers are shown in Table 5.

**Table 5:** Cumulative pregnancy results per total transfer in MPA and GnRH antagonist groups.

Variables		GnRH antagonist(n = 584)	MPA (n = 519)	*P* value
ositive hCG (%, n)	None	%60,3 (352)	%55,3 (287)	$0{,102}^{\text{a}}$
	Yes	%39,7 (232)	%44,7 (232)	
Biochemical pregnancy (%, n)	None	%90,8 (530)	%90,6 (470)	$0{,919}^{\text{a}}$
	Yes	%9,2 (54)	%9,4 (49)	
Clinical pregnancy (%, n)	None	%69,9 (408)	%64,7 (336)	$0{,077}^{\text{a}}$
	Yes	%30,1 (176)	%35,3 (183)	

^a^Pearson chi-square.

According to the table, no statistically significant differences were found in the total hCG positivity rate (*p* = 0.102), biochemical pregnancy rate (*p* = 0.919), or cumulative clinical pregnancy rate *(p* = 0.077) between the MPA and GnRH antagonist groups. The cumulative clinical pregnancy rate per total transfer was 35.3% in the MPA group and 30.1% in the GnRH antagonist group.

## Discussion

In our study, a premature LH surge was defined as a 1.8-fold increase in basal LH values during the OS cycle. There was no significant difference in the occurrence of a premature LH surge between the MPA and GnRH antagonist groups (*p* = 0.708). The rate of premature LH surge in the MPA group (19.3%) was lower than that in the GnRH antagonist group (20.1%). The frequency of premature ovulation was similar between the GnRH antagonist (2.2%) and MPA (2.9%) groups (*p* = 0.415).

There were no significant differences in the clinical pregnancy rate (p = 0.074) between the two groups after the first ET, in the cumulative clinical pregnancy rates per total transfer (*p* = 0.077), or in the cumulative pregnancy results after the transfer of all embryos obtained from a single cycle (*p* = 0.705).

A premature LH surge during OS is an important cause of cycle cancellation in IVF treatment. GnRH analogues are frequently used to prevent premature LH surges^2^. However, GnRH analogues have disadvantages such as increased risk of OHSS, high cost, and procedure complexity^3^. The search for new methods to prevent premature LH surges is still ongoing.

In the literature, progestins have been considered to be a suitable alternative to conventional GnRH analogue therapy for OS^4^. In recent years, when frozen-thawed ET was not widely used, progestins were not considered for use in patients with planned fresh ET because they are known to have a negative effect on endometrial receptivity. However, with advances in embryo vitrification technologies, frozen-thawed ETs are increasingly being performed by clinicians interested in reproductive endocrinology worldwide.

In addition, freezing all embryos in cases of excessive response may avoid OHSS, which may pose a life-threatening risk for patients. Therefore, synthetic progestins can be used to prevent premature LH surges in IVF cycles via a freeze-all approach due to their efficacy in LH suppression^7^.

In our centre, MPA is frequently preferred as an oral progestin for the suppression of premature ovulation. Despite its moderate/strong progesterone effect, MPA has fewer androgenic effects^9^. When using MPA, endogenous progesterone can be measured during OS and allows for early luteinization detection. For these reasons, the use of MPA in IVF practice is frequently preferred by physicians. Studies have shown that 10 mg of MPA is effective in preventing premature LH surge and ovulation^10^.

In our study, MPA was used at a dose of 10 mg. Contrary to the literature, LH levels were found to be higher both during stimulation and on the day of triggering in patients using MPA. However, although a higher daily initial FSH dose was used in patients using MPA, the total gonadotropin dose used was lower due to shorter stimulation times. This may be because clomiphene citrate (CC) was mostly used simultaneously with MPA in our centre. Therefore, CC may cause an increase in pituitary-derived endogenous gonadotropins. As a result, it is possible that the suppression of pituitary-derived endogenous gonadotropins due to progestins was compensated for by the stimulatory effect of CC. In our study, Clomiphene Citrate was used %30,3 in GnRH antagonist group and %35,6 in MPA group and there was no significant difference between two groups (*p* = 0,122). By using MPA in combination with CC, both the cost of OS treatment may be reduced, and patients may receive treatment for a shorter period of time^11^.

GnRH analogues are known to have higher unit costs than oral progestins. Choosing progestins for the prevention of premature ovulation will result in a significant reduction in treatment costs. However, it is still unclear whether they are cost-effective because of the mandatory need to freeze all embryos in cycles in which progestins are used and the already significant cost of these procedures. Recent studies suggest that the "freeze-all" approach may be cost-effective if pregnancy rates increase by approximately 6–11%^12^. However, since there are differences in both drug and IVF treatment costs between countries, there is still a need for cost-effectiveness studies.

There are concerns that oocyte and embryo quality may be adversely affected in cycles in which progestins are used^13^. However, recent studies have shown that the use of MPA has no adverse effect on oocyte quality, fertilisation, or embryo development rates^7^. In our study, the total number of oocytes obtained was significantly lower in the MPA group than in the GnRH antagonist group. However, the mean number of antral follicles was also significantly lower in the MPA group than in the GnRH antagonist group. Therefore, patients with a worse prognosis in terms of treatment success may have been those in the MPA group. On the other hand, the number of MII oocytes obtained was similar between the groups. In this context, although the number of oocytes obtained was lower, there was no difference in the number of mature oocytes. Therefore, the percentage of maturation of the obtained oocytes was greater in patients who used MPA. In addition to the number of developing embryos, embryo morphology is moderately correlated with the euploidy rate. High-quality embryos with high euploidy ratios may increase healthy live birth rates^14^. In our study, we found that the number of high-quality embryos that developed was greater in patients who received MPA. In conclusion, the oocyte maturation rate and the number of high-quality embryos per mature oocyte obtained may be greater in patients treated with MPA.

It has been shown that the use of MPA can effectively prevent premature LH surges during OS without adversely affecting embryo development^5,7^. However, there is no consensus on the definition of premature LH surge in the literature. A maximum LH level above 10 mIU/mL or 15 mIU/mL during the cycle or a 1.8-fold increase compared to the basal level has been accepted as an LH surge in different studies in the literature^15^. In our study, a separate analysis was performed for each of the definitions of LH surge in the literature. First, when a premature LH surge was accepted as a 1.8-fold increase in LH compared to the previous measurement, there was no significant difference in the incidence of premature LH surges between the two groups. Therefore, when a 1.8-fold increase in the serum LH level compared to the previous measurement was accepted as the definition, MPA was found to effectively prevent premature LH increase. However, the frequency of peak LH values of 15 mIU/mL and above was found to be significantly greater in patients receiving MPA. The frequency of peak LH levels of 15 mIU/mL and above was 6.1% in the MPA group and 3.6% in the GnRH antagonist group. Similarly, the frequency of peak LH levels of 10 mIU/mL and above during stimulation was found to be significantly greater in the MPA group. Therefore, if an LH level of 10 mIU/mL or 15 mIU/mL or above during the cycle is considered a premature LH surge, MPA may not be as effective as GnRH antagonists. On the other hand, during OS, the aim is to prevent premature ovulation, not premature LH surge. In this context, premature ovulation was observed with a similar frequency in patients receiving both GnRH antagonists and MPA (2.2% vs. 2.9%). In conclusion, although higher LH levels are encountered during OS with the use of MPA, premature ovulation can be prevented with similar efficacy with both MPA and GnRH antagonists.

In our study, 61.6% of the patients in the MPA group and 70.6% in the GnRH antagonist group underwent at least one ET. Therefore, in approximately one-third of the patients, ET could not be performed due to the lack of embryo development to be transferred, and the treatments of the patients were cancelled.

Progestins are known to have negative effects on endometrial receptivity^16^. Therefore, fresh ET was not performed in any of the patients using MPA in our study, and all developing embryos were frozen. In contrast, both fresh and frozen ETs can be performed in patients in whom GnRH antagonists are used. In patients with a high response during OS, a risk of OHSS, high progesterone levels on the day of triggering, endometrial polyp, or myoma uteri-like pathologies that may prevent implantation during stimulation, or tubal-induced hydrosalpinx, ETs may not be performed, and all developing embryos may be frozen. Consistent with this, total freezing was performed in 39.8% of the patients in our study in whom GnRH antagonists were used.

Cumulative pregnancy rates obtained by transferring all embryos that developed after a single OS may be a more appropriate outcome variable for evaluating the success of the treatment than pregnancy rates per ET^17^. In our study, no difference was found between the cumulative clinical pregnancy rates after all ETs were performed in either group. However, since the patients in the MPA group had been treated more recently, it is still possible that they had already frozen embryos but had not yet applied to the centre for ET. Therefore, we evaluated clinical pregnancy rates after the first ET and cumulative pregnancy results per total transfer. There was no difference between the two groups (*p* = 0.074, *p* = 0.077). Nevertheless, it can be said that the cumulative pregnancy rate associated with MPA is at least similar to that associated with GnRH antagonists.

The most important limitations of our study are its retrospective design, and we did not evaluate the effects of CC. Another limitation of our study is that we did not evaluate the variables of patients by the final triggering type.

There is still a need for prospective randomized controlled trials to determine the optimal doses of MPA for use in treatment protocols to verify its efficacy and safety and to demonstrate its effects on oocyte development.

In conclusion, the use of MPA during OS can effectively prevent premature ovulation. The OS, embryo development and pregnancy outcomes of patients receiving MPA were similar to those of patients receiving GnRH antagonists. Therefore, the use of MPA instead of GnRH antagonists may be an effective alternative for patients for whom fresh ET is not planned.

## Data Availability

The data of this study are available at the Department of Gynecology and Obstetrics, Akdeniz University. For the data of this manuscript you may contact Prof. Dr. Şafak OLGAN and Dr. Can DİNÇ. E mail adresses are; safakolgan@gmail.com candinc00@hotmail.com.
